# A Case of Terson-Like Syndrome in a Patient with Viral Meningoencephalitis

**DOI:** 10.1155/2019/9650675

**Published:** 2019-04-24

**Authors:** Masumi G. Asahi, Stephanie J. Weiss, Krishi Peddada, Deepika Malik

**Affiliations:** ^1^Western University of Health Sciences, 309 E 2nd St., Pomona, CA 91766, USA; ^2^Drexel University College of Medicine, 219 N. Broad St., 3rd Floor, Philadelphia, PA 19107, USA; ^3^Weill Cornell Medical College, 1305 York Ave., New York, NY 10021, USA; ^4^Moore Eye Institute, 100 W Sproul Rd., Springfield, PA 19064, USA; ^5^Philadelphia College of Osteopathic Medicine, 4170 City Ave., Philadelphia, PA 19131, USA

## Abstract

The proposed mechanism of Terson's syndrome is increased intracranial pressure that leads to dilation of the retrobulbar optic nerve and compression of the central retinal vein. Terson's syndrome has been associated with many conditions that increase intracranial pressure such as venous sinus thrombosis, Moyamoya disease, leukemia, direct head trauma, and intraocular hemorrhage related to shaken baby syndrome. We present a novel case of a patient with recent viral prodrome found to have papilledema and multilayered retinal hemorrhages consistent with Terson syndrome. Computed tomography and magnetic resonance venography of the brain did not reveal any subdural, subarachnoid, or intracranial hemorrhages. However, cerebrospinal fluid analyses were significant for increased opening pressure and elevated protein levels, which were suggestive of viral meningoencephalitis. We describe this case as a Terson-like syndrome because the etiology of intraocular hemorrhage is increased intracranial pressure. However, this case does not fit the traditional presentation of Terson's syndrome as the intracranial pressure is secondary to meningeal inflammation instead of subdural, subarachnoid, or intracranial hemorrhage. We strongly feel that it is important for physicians to be aware of the link between viral meningoencephalitis and retinal conditions such as Terson-like syndrome because it can facilitate rapid diagnosis and treatment.

## 1. Introduction

In 1881, German ophthalmologist Moritz Litten described vitreous bleeding occurring with subarachnoid hemorrhage, a condition that later became named Terson's syndrome. The cause was presumed to be subarachnoid blood being transmitted directly forward through the optic nerve sheath [1]. More recently, Terson's syndrome has been understood in the context of increased intracranial pressure resulting from any number of conditions that cause subdural, subarachnoid, or intracranial hemorrhage. Some of the conditions associated with Terson's syndrome include venous sinus thrombosis, Moyamoya disease, leukemia, direct head trauma, and intraocular hemorrhage related to shaken baby syndrome [2–6]. In Terson's syndrome, increased intracranial pressure causes rapid effusion of cerebrospinal fluid into the optic nerve sheath [1, 7], which in turn results in dilation of the retrobulbar optic nerve and subsequent compression of the central retinal veins [1, 7]. The venous obstruction causes venous stasis, venous hypertension, and distension, ultimately resulting in rupture of thin retinal vessels and subsequent hemorrhage [1, 2, 7].

Viruses can cause central nervous system (CNS) infections and contribute significantly to the disease burden in humans globally [8]. While most viral CNS infections are benign and self-limiting, some can cause severe inflammation and result in severe long-term neurologic dysfunction [8]. Viral meningoencephalitis is typically associated with fever, headache, photophobia, and neck stiffness whereas viral encephalitis is associated with fever, seizures, focal neurologic deficits, and decreased consciousness [9]. Thus far, the only reported ocular complications linked to viral meningoencephalitis include sixth nerve palsy, optic neuritis, and papilledema [10–12]. We describe a case of viral meningoencephalitis with multilayered retinal hemorrhages suggestive of a Terson-like syndrome.

## 2. Case Presentation

**Figure 1:**
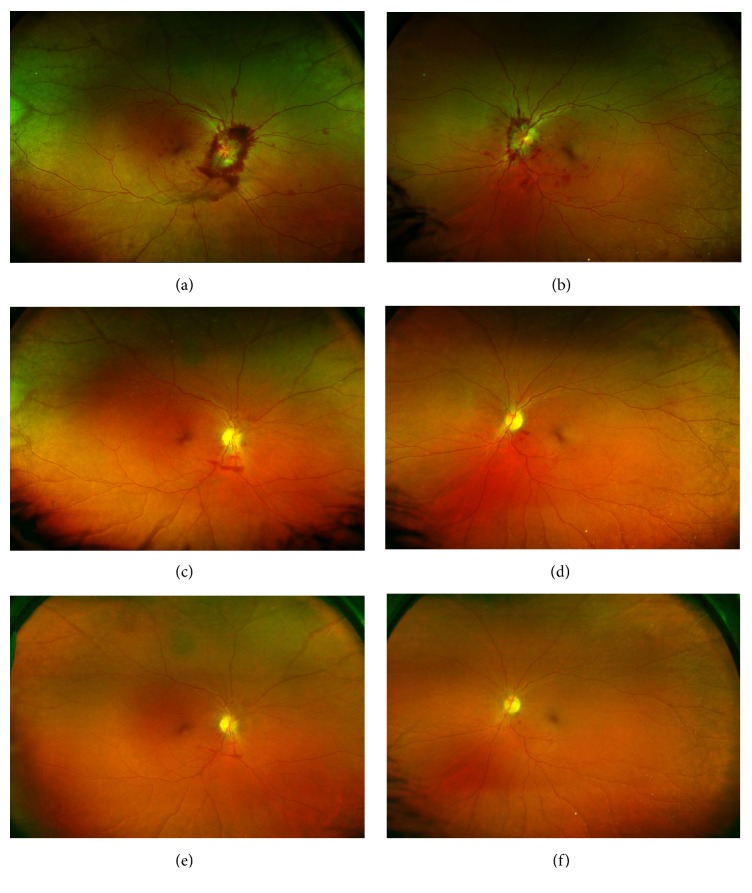
Optos Fundus Photos showing (a) right eye and (b) left eye at presentation showing preretinal hemorrhages, flame hemorrhages, and intraretinal blot hemorrhages with optic disc edema consistent with Terson syndrome. (c, e) Right eye and (d, f) left eye showing disc pallor consistent with optic atrophy and resolving hemorrhages with persistent inferior preretinal hemorrhages inferior to the disc at (c, d) 3-month follow-up and at (e, f) 4-month follow-up.

**Figure 2:**
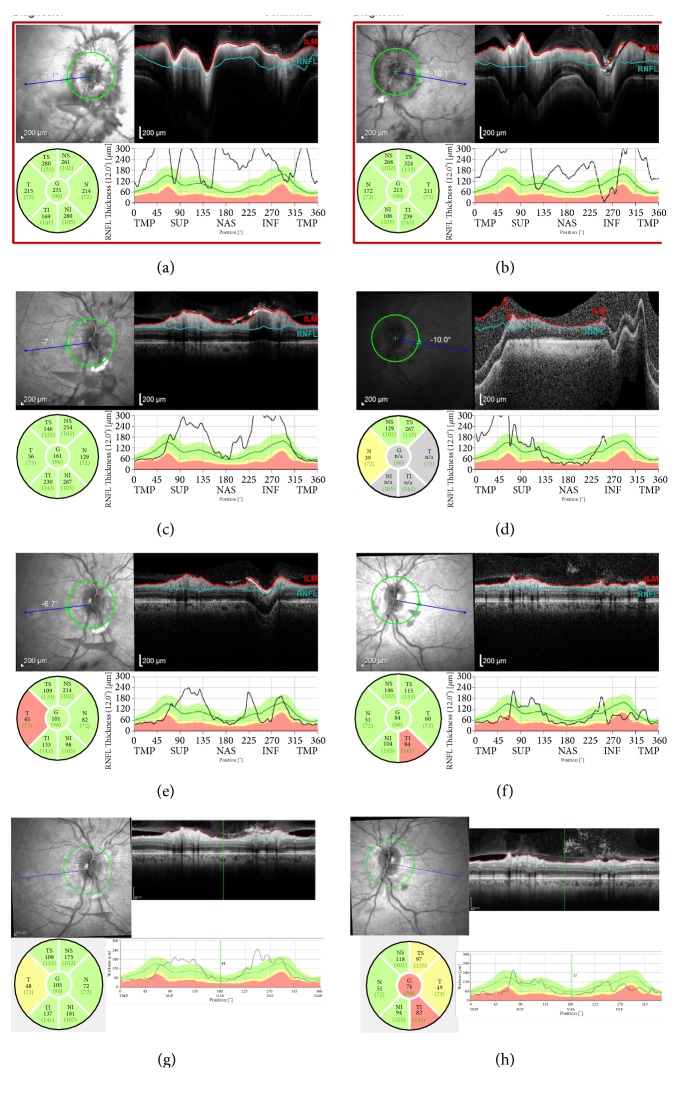
Retina Nerve Fiber Layer (RNFL) Optical Coherence tomography (OCT) of the (a, c, e, g) right eye and (b, d, f, h) left eye showing (a, b) disc edema at presentation, (c, d) improving disc edema at six-week follow-up, and (e, f) near resolution of disc edema at twelve weeks and (g, h) sixteen weeks. Sectoral thickness measured in microns, and thickness of cross-section in temporal (TMP), superior (SUP), nasal (NAS), and inferior (INF) graphed against normative population data set.

**Figure 3:**
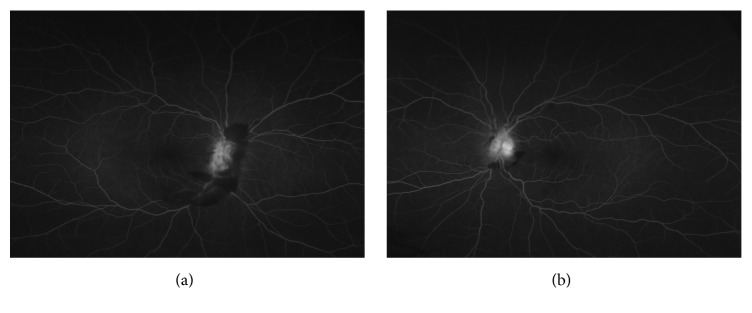
Fluorescein angiography (FA) taken at six minutes of (a) right and (b) left eye showing disc hyperfluorescence without disc leakage and surrounding peripapillary hemorrhages.

A 50-year-old African American female presented with a three-week history of intractable headache, confusion, vertical binocular diplopia, photophobia, and difficulty with balance. Examination revealed best corrected visual acuities of 20/25 in the right eye (OD) and 20/20 in the left eye (OS) with intraocular pressures (IOP) of 14 mmHg in both eyes (OU). No afferent pupillary defect was noted, extraocular motility (EOM) was full OU, and confrontational visual fields were full OU. Anterior segment examination in both eyes was unremarkable. Funduscopic examination of both eyes revealed grade-four optic disc edema, blurring of optic disc margin, and obscuration of vasculature of the optic nerve. Preretinal and intraretinal hemorrhages were present in both eyes, largely concentrated in the peripapillary retina, but extending to the mid-peripheral retina (Figures 1(a) and 1(b)). Optic nerve swelling was confirmed on optical coherence tomography (OCT) of the retinal nerve fiber layer (Figures 2(a) and 2(b)). Fluorescein angiography revealed hyperfluorescence of the optic disc OU (Figures 3(a) and 3(b)).

Computed tomography of the head, magnetic resonance imaging of the brain, and magnetic resonance venography of the brain were unremarkable and had no evidence of intracranial mass, hemorrhage, or ischemia. Cerebrospinal fluid (CSF) analysis revealed an opening pressure of 30 cm H_2_O, 77 white blood cells/mm^3^ with 94% lymphocytes, 93 mg/dL protein, and 73 mg/dL glucose. Bacterial and fungal cultures of the CSF were negative. CSF studies for Lyme, syphilis, herpes simplex virus, and West Nile virus were unremarkable. A limited panel consisting of laboratory studies for enterovirus, syphilis, ANA, and p-ANCA was negative. A respiratory viral panel for adenovirus, cytomegalovirus, influenza, parainfluenza, rhinovirus, and respiratory syncytial virus was negative on Polymerase Chain Reaction testing. Based on the patient's presentation and cerebrospinal fluid findings, she was diagnosed with viral meningoencephalitis. She was monitored as an inpatient with conservative management. During the hospital course, the patient noted improvement in her double vision and headaches. A repeated lumbar puncture 5 days later revealed opening pressure of 4 cm H_2_O, 38 white blood cells/mm^3^, 75 mg/dL protein, and 57 mg/dL glucose. The patient was subsequently discharged from the hospital.

**Figure 4:**
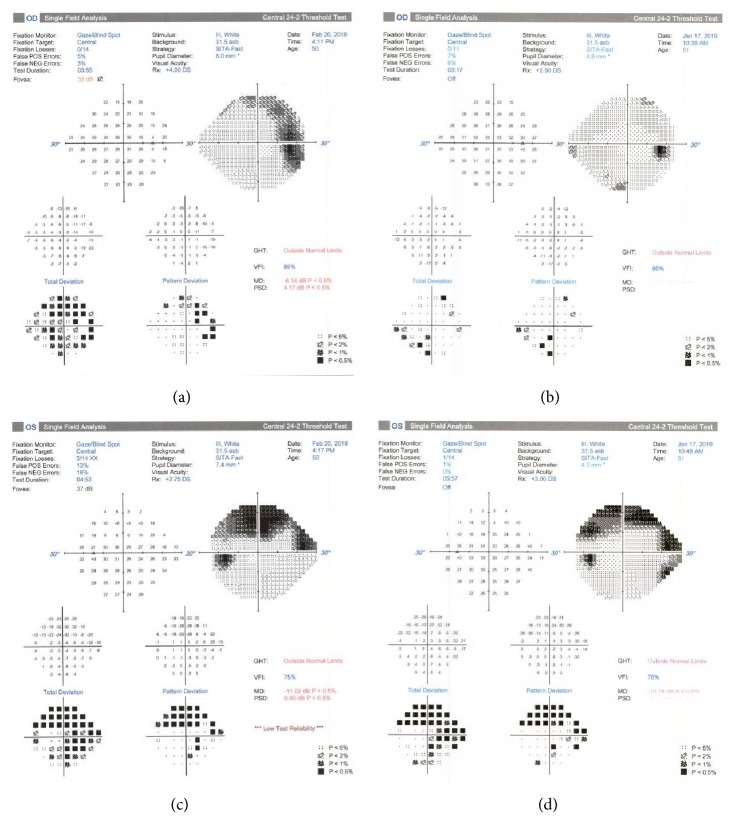
Humphrey visual field of the patient of the right eye shows scattered deficits both upon presentation (a) and almost one year later (b). A dense superior arcuate defect seen in the left eye on presentation (c) is consistent and has not progressed almost one year later (d). This superior arcuate defect corresponds to inferotemporal thinning of the retinal nerve fiber layer on OCT of the left eye.

Follow-up examinations with serial OCT of the optic nerve showed continued progressive improvement in disc edema (Figures 2(a)–2(h)) along with complete resolution of papilledema and peripapillary hemorrhages (Figures 1(a)–1(f)). Mild residual pallor of both optic nerves was observed at the end of one year. OCT revealed borderline temporal thinning of the right optic nerve. The left optic nerve had borderline temporal thinning, borderline superotemporal thinning, and inferotemporal thinning. The findings of persistent disc pallor and inferotemporal nerve fiber layer thinning on OCT were consistent with the persistent visual field deficit documented on Humphrey visual fields (Figures 4(a)–4(d)).

## 3. Discussion

This case report presents a patient in whom increased intracranial pressure was associated with hemorrhage from the peripapillary retinal vessels [13]. Based on the papilledema and peripapillary hemorrhages, we hypothesize that a Terson-like mechanism of increased intracranial pressure led to dilation of the central retinal veins and subsequent retinal hemorrhaging. The mechanism of increased intracranial pressure was presumed to be due to inflammation from viral meningoencephalitis. In encephalitis, inflammation is a key part of the pathway as cytokines increase permeability of blood vessels and leak plasma proteins into the CSF [13]. The subarachnoid exudates of proteinaceous material and leukocytes can diminish the resorptive capacity of arachnoid granulations and obstruct CSF flow, leading to hydrocephalus and interstitial edema [13]. The combination of vasogenic and cytotoxic edema can result in elevated intracranial pressure in patients with viral meningoencephalitis. This case is an important addition to the literature because it provides an etiology for some of the approximately 5.3% of patients with meningitis or meningoencephalitis with retinal hemorrhages [14]. Such hemorrhages have been described in a wide range of CNS infections ranging from West Nile Virus to Rickettsia [15, 16].

Our patient was managed conservatively, as intraretinal hemorrhages tend to resolve spontaneously in most cases [17]. Vision typically improves with resolution of the intraretinal hemorrhages, but permanent vision impairment can occur. In patients with persistent vitreous hemorrhage or premacular hemorrhage, pars plana vitrectomy has been shown to be effective treatment [18]. One must carry a high degree of suspicion for Terson's syndrome, and we recommend early fundoscopic screening in cases of visual disturbances with unknown etiology. Although this case was not a classic presentation of Terson's syndrome, it may be possible that Terson's syndrome itself may be associated with a wider spectrum of neurological disease entities and is more common than previously thought.
